# Does the Cambridge classification alone predict the possibility to perform echocardiographic examination in lateral recumbency in dogs affected by brachycephalic obstructive airways syndrome?

**DOI:** 10.1371/journal.pone.0286914

**Published:** 2023-06-07

**Authors:** Mara Bagardi, Chiara Locatelli, Sara Ghilardi, Federica Creta, Beatrice Pasquinelli, Paola G. Brambilla, Stefano Romussi

**Affiliations:** Department of Veterinary Medicine and Animal Sciences – DIVAS, University of Milan, Lodi (LO), Italy; Universiti Malaya Fakulti Perubatan: University of Malaya Faculty of Medicine, MALAYSIA

## Abstract

**Objectives:**

To evaluate if the functional grading system (Cambridge classification) of brachycephalic obstructive airways syndrome (BOAS) and the temperament score can be useful tools in predicting the feasibility of echocardiographic examination in lateral recumbency. The hypothesis is that the temperament of the dog, rather than the severity of BOAS alone, can exacerbate respiratory symptoms (dyspnea, stertor, stridor and/or cyanosis) during lateral containment.

**Methods:**

Prospective cross-sectional study. Twenty-nine French Bulldogs were included and classified according to the Cambridge classification for the BOAS and to the Maddern score for the temperament. Receiver operating characteristic analysis was used to evaluate the sensitivity (Se) and specificity (Sp) of the Cambridge classification, of the temperament score and their sum to predict the feasibility of the echocardiography in lateral recumbency without dyspnea/cyanosis.

**Results:**

8 females (27.59%) and 21 (72.41%) males French Bulldogs of 3 years (IQR25-75 1–4), and 12.45 kg (IQR25-7511.5–13.25) were included. The Cambridge classification alone was not predictive for the possibility of performing the echocardiography in lateral recumbency, unlike temperament score and the sum of the two classification indices. The diagnostic accuracy of Cambridge classification (AUC 0.81, Se 50%, Sp 100%), temperament score (AUC 0.73, Se 75%, Sp 69%), and their sum (AUC 0.83, Se 75%, Sp 85%) cut-offs was moderate for each score.

**Clinical significance:**

The dog’s temperament, and therefore its susceptibility to stress, rather than the severity of BOAS (Cambridge classification) alone, is a good predictor of the possibility of performing the echocardiographic examination in standing instead of lateral recumbency.

## Introduction

The canine Brachycephalic Obstructive Airway Syndrome (BOAS) is a set of pathological conditions affecting the upper airways, with a morphological and functional basis, linked to the combination of congenital anatomical abnormalities typical of brachycephalic breeds. Among the most affected breeds, the English Bulldog, the French Bulldog (FB), the Pug, the Boston Terrier, and the Pekingese are the most represented [1].

Due to the growing popularity acquired by these breeds over the past two decades and the consequent increase in recorded cases of BOAS, awareness of how this syndrome constitutes a major health and well-being problem has increased [2]. To date there are several published studies, and it is now clear that the list of problems of brachycephalic breeds does not only concern the respiratory system, but also involves numerous other districts [3–7].

The classification of the respiratory condition of the brachycephalic dogs, however, is not yet univocal. An objective method useful to make a diagnosis, to determine the severity of the disease, to base the therapeutic choice and therefore to minimize the impact of the pathology on the well-being of the animal is needed and is still very much of interest for clinicians and surgeons who approach these problems.

Several brachycephalic patient assessment approaches have been proposed over the years [2,8–12], as well as several notions regarding the alterations resulting from the genetic selection carried out in the brachycephalic breeds. However, there is not a gold standard diagnostic test, and the clinician experience remains essential [2]. An appropriate evaluation of patients affected by BOAS should include at least a neck and chest X-ray and an endoscopic airway assessment [6], procedure that obviously require pharmacological containment.

The Cambridge classification [2,10,12] is currently the only accredited and most widely used for the diagnosis and classification of subjects affected by BOAS. It is an objective and non-invasive method obtained by combining the results of several tests such as the functional grading system, the whole-body barometric plethysmography, the determination of the tidal breathing flow volume loops, the head-out whole-body plethysmography, and the spirometry [10,13–16].

The purpose of this study was to evaluate the predictivity of the Cambridge classification for performing collateral diagnostic procedures. In particular, the echocardiographic examination may be considered as a useful collateral diagnostic procedure for the pre-surgical evaluation of dogs suffering from BOAS and for breeding cardiological screening. In fact, these dogs are frequently very difficult to auscultate, especially before a BOAS’ corrective surgery [17]. Chronic, obstructive respiratory diseases, such as BOAS, can influence the cardiovascular system and may result in secondary changes [18]. Echocardiography is the most useful diagnostic tool for the evaluation of anatomy and cardiac function in the clinical practice of small companion animals [19,20] and is therefore important to exclude pathologies and cardiac abnormalities to which brachycephalic breeds are particularly predisposed such as, for example, pulmonary valve stenosis.

In clinical practice, the echocardiographic examination is normally performed with the dog in lateral recumbency (both right and left), regardless of size. This positioning, in fact, reduces the interference of the lungs, allows a single operator to easily contain the patient, facilitates the collection of M-mode, B-mode and Doppler data and limits the measurements’ variability due to the different height of the dogs in a standing position [21]. On the other hand, lateral recumbency containment can be a source of stress and/or agitation for the animal and the non-compliance of the patient can affect the results of the examination [21]. Within-day variability of conventional echocardiography performed with the dog in the standing position was at least as good as that obtained with the dog in lateral recumbency for most measured variables. Single measurements of each variable may be sufficient for trained observers examining dogs that do not have an arrhythmia. The standing position should be used, particularly for stressed or dyspneic dogs [21]. It has been noted that in the brachycephalic dog, particularly if agitated, some postural attitudes modify the anatomy of the upper airways, especially those parts without bone support on which the gravity given by the position already acts, with consequent exacerbation of any respiratory deficits and manifestation of symptoms, such as dyspnea or cyanosis [22], making it extremely difficult to perform, forcing the cardiologist to perform the examination in standing position.

A recent study conducted by Bagardi et al. (2022) compared the echocardiographic measurements obtained both in lateral recumbency and standing position in 40 French Bulldogs and the obtained results with the data currently available in the literature [21,23–25]. This study demonstrated that there are no measurements’ differences between the two positioning, according to the previous studies [21,24,25]. The data obtained by Bagardi et al. may not be applicable in dogs with heart diseases and in other breeds due to the different chest morphology [23].

The present study aims to evaluate if the functional grading system of the Cambridge classification and the temperament score can be useful tools in predicting the feasibility of echocardiographic examination in lateral recumbency in FB affected by BOAS. It has been hypothesized that the temperament of the dog, and therefore its susceptibility to stress, rather than the severity of BOAS (Cambridge classification) alone, can exacerbate respiratory symptoms (dyspnea, stertor, stridor and/or cyanosis) during lateral containment. If this hypothesis is true, the Cambridge classification may be considered non-exhaustive for the overall definition of the brachycephalic patient’s respiratory condition.

## Materials and methods

This prospective diagnostic performance study included owned FB referred to the University Veterinary Teaching Hospital of Lodi–University of Milan–between July 2020 and May 2022 for BOAS evaluation/staging and possible surgical correction. The study included adult dogs (from 1 to ten years) not affected by acquired or congenital diseases other than BOAS. For each patient, age, gender, weight, and body condition score (BCS) (WSAVA’s Global Nutrition Guidelines, 2011) were considered. Every echocardiographic examination was made as part of a diagnostic process for the evaluation of brachycephalic patients and every owner signed a written informed consent before enrollment. Owners were informed that data obtained would be used for our research and all the owners accepted it. Informed consent was obtained according to the Institutional Ethics Committee of the University of the Study of Milan (http://www.unimi.it/ateneo/20138.htm).

All patients included in the study were evaluated according to the functional grading system developed by the Cambridge University group (Cambridge classification) [2,10]. Each patient was then classified in one of the three degrees of severity of BOAS following the evaluation of respiratory symptomatology before and after a 3-minute exercise tolerance test at a trot at a speed of 4–5 km/h [10] (Table 1).

**Table 1:** Functional grading system of the Brachycephalic obstruction airway syndrome (BOAS) developed by the Cambridge University group.

Score	Description
**0**	Free of respiratory signs; annual check is suggested if dog is under 2 years old.
**1**	Mild respiratory signs of BOAS but does not affect exercise tolerance. Annual health check is suggested if the dog is under 2 years old.
**2**	Moderate respiratory signs of BOAS. The dog has a clinically relevant disease and requires managements, including weight loss and/or surgical intervention.
**3**	Severe respiratory signs of BOAS. The dog should have a thorough veterinary examination with surgical intervention.

Each dog was also evaluated for temperament according to the Maddern score [26] (Table 2).

**Table 2:** Temperament score proposed by Maddern et al., 2010 [26].

Score	Description
**1**	Calm with playful attitude
**2**	Slightly nervous and/or agitated
**3**	Moderately nervous and/or agitated
**4**	Very nervous and/or agitated

Following clinical evaluation, each patient underwent a complete echocardiographic examination [27]. For each patient were evaluated the feasibility of echocardiographic examination in lateral recumbency, by a cardiologist (MB) blinded to Cambridge and temperament score, and the presence of variation of the respiratory condition between the containments. In the lateral recumbency, were evaluated: need of manual containment; worsening of respiratory clinical signs with the onset of polypnea, dyspnea, stridor, stertor or cyanosis; postural changes in the neck and trunk region; contraction response of the sternocleidomastoid muscles; general contraction response of the musculature with the possible appearance of tremors; possible presence of urination and/or contraction of the sphincter with expression of the anal sacs. For each patient, a standard bipolar (I, II and III) and unipolar (aVR, aVL, and aVF) electrocardiogram (ECG) was performed to detect any cardiac arrhythmias or conduction disorders. The chest wall area was trichotomized in all dogs at 4th-6th intercostal space both on the right and on the left. Echocardiographic studies were carried out using a portable Doppler ultrasound device (Esaote, MyLabTM Omega, Genova, Italy) with multifrequency probes (1–4 MHz, 3–11 MHz) and a simultaneous electrocardiographic trace. No dog was sedated during the procedure.

The Cambridge Classification and the temperament scores were carried out by a well-trained single operator (FC). The echocardiographic examination was also carried out on all patients by a single cardiologist (MB).

### Statistical analysis

Statistical analyses were performed by a veterinary cardiology specialist clinician (MB) with statistics training using commercially available statistics software (SPSS 27.0, IBM, SPSS, USA; MedCalc Statistical Software version 19.3.1, MedCalc Software Ltd, Ostend, Belgium; https://www.medcalc.org; 2020). Descriptive statistics were generated. The distribution of data for continuous variables was assessed for normality by means of the Kolmogorov-Smirnov test. None of the variables were normally distributed, and results were reported as the median and interquartile range (IQR) (25th to 75th percentile) unless otherwise specified.

The Mann-Whitney U nonparametric test was performed to assess whether the distribution of age, weight, BCS, Cambridge classification (grade) and temperament score was the same among sex categories. The Spearman rank-order correlation coefficient (rS) was calculated to determine the strength of the association between the Cambridge classification and the temperament score with age, weight and BCS. The correlation was considered weak, moderate, strong, or perfect, respectively, when the value of the correlation coefficient was .1-.3, .4-.6, .7-.9, or 1 [28]. The Fisher’s exact test was used to determine if there were non-random association between two categorical variables and to so if the temperament of the dog, rather than the severity of BOAS alone, can exacerbate respiratory symptoms during lateral containment.

Receiver operating characteristic (ROC) analysis was used to evaluate the sensitivity and specificity of the Cambridge classification and of the temperament score to predict the feasibility of the echocardiography in lateral recumbency. The optimal clinically relevant cutoff value for both classifications and for their sum to predict the possibility to perform the echocardiographic examination in lateral recumbency was determined using the Youden index. The area under the ROC curve (AUC) was used to assess the diagnostic accuracy and quantify the predictive value of Cambridge classification and temperament score: AUC = 0.5, the test is not informative; 0.5 < AUC ≤ 0.7, the test is inaccurate; 0.7 < AUC ≤ 0.9, the test is moderately accurate; 0.9 < AUC < 1.0, the test is highly accurate; AUC = 1, perfect test [29].

P-values < 0.05 were considered significant for all analyses.

## Results

Twenty-nine FB were included, 8 females (27.59%) and 21 (72.41%) males. Clinical data were collected for all patients. The median age was 3 years (IQR25-75 1–4), and the median weight was 12.45 kg (IQR25-75 11.5–13.25).

A Cambridge classification score was assigned [10], as well as a temperament score (Table 3a) and their sum (Table 3b).

**Table 3:** a. Distribution of all included dogs for each Cambridge and temperament scores. b. Distribution of all included dogs for the sum of Cambridge and temperament scores.

Cambridge Classification			Temperament score			Cambridge Classification + Temperament score		
Grade	Number of dogs	Percentage of dogs	Score	Number	Percentage	Score	Number	Percentage
**0**	6	20.9	**1**	9	31.1	1	4	13.8
**I**	4	13.8	**2**	5	17.2	2	5	17.2
**II**	12	41.4	**3**	7	24.1	3	4	13.8
**III**	7	24.1	**4**	8	27.6	4	5	17.2
						5	4	13.8
						6	4	13.8
						7	3	10.4
						1	4	13.8

The echocardiographic examination was performed only in standing position in 14 dogs (48.3%).

In all these patients the inability to perform the echocardiographic examination in lateral recumbency was linked to the appearance of polypnea and worsening of stertor symptoms and to the appearance of postural changes in the neck and trunk region with contraction of the sternocleidomastoid muscles and general contraction muscle with the onset of tremors. Of these 14 dogs, 6 (42.9%) experienced the onset of dyspnea when positioned in lateral recumbence, 5 (35.7%) cyanosis, 1 (7.1%) stridor and 2 (14.3%) sphincter contraction with expression of the anal sacs. There were no differences of age (p = 0.978), weight (p = 0.354), BCS (p = 0.238), Cambridge classification (p = 0.218) and temperament score (p = 0.374) between males and females.

There were no significant correlations between Cambridge classification, temperament score and their sum and age, weight and BCS (p > 0.05). The correlation between Cambridge classification and temperament score is positive and weak (rs = 0.34), but not significative (p = 0.097).

According to Fisher’s exact test, the Cambridge classification alone was not able to predict the execution of the echocardiographic examination in standing or lateral recumbency (p = 0.003), unlike the temperament score and the sum of the two classification indices (p = 0.164 and p = 0.134, respectively).

The diagnostic accuracy of optimal Cambridge classification, temperament score, and their sum cut-offs for prediction of the possibility of performing the echocardiographic examination in lateral recumbency was reported, as well as sensitivity and specificity of each method (Table 4). Values greater than the cut-offs correspond to the impossibility to perform the examination in lateral recumbency.

**Table 4:** Diagnostic accuracy of Cambridge classification, temperament score, and their sum cut-offs for prediction of the feasibility of echocardiography in lateral recumbency.

Variable	AUC	p value	95% CI	Cut-off	Sensitivity %	Specificity %
**Cambridge classification (A)**	0.81	0.00	0.64–0.98	2.50	50	100
**Temperament score (B)**	0.73	0.03	0.52–0.93	2.50	75	69
**A+B**	0.83	0.00	0.67–0.99	4.50	75	85

AUC: Area under the curve; CI: Confidence interval.

## Discussion

The Cambridge classification has been extensively studied on healthy and affected by BOAS dogs belonging to three different breeds (Pug, FB, English Bulldog). It has been the only one widely discussed by literature about the advantages and limits of its use and, the only one that obtained the consensus [9,18,30–32]. Furthermore, this classification has been the only one proposed as an objective method of evaluating surgical results [31], basing on the owner perception. However, 60% of owners of affected dogs are not able to recognize the BOAS clinical signs, which can cause a delay in treatment and further deterioration of the disease, reporting in their medical history untruthful data [33].

The FB is one of the breeds most predisposed to BOAS. However, a proportion of FB who are exposed to the risk of having BOAS (i.e., extreme brachycephalic skull dimensions) do not develop respiratory signs that are clinically concerning until they are stressed or forced into uncomfortable positions, such as the lateral recumbency.

For these reasons, this is the first study which evaluates the predictivity of the Cambridge classification and of the temperament score on the possibility of performing collateral diagnostic procedures (echocardiography) in FB and of supporting stressful conditions (lateral recumbency). This type of containment causes an alteration of the respiratory tract anatomy due to gravity, especially for those parts without anatomical support, such as the pharynx. This positioning, moreover, is generally poorly tolerated by patients, who, if agitated, assume postural changes of the neck and trunk due to contraction of the sternocleidomastoid muscles with consequent shortening of the neck visceral space, compression of the soft tissues and decrease of the free space available for air passage. Consequently, there is a modification of the respiratory conditions with exacerbation of any respiratory deficits and the appearance of polypnea, dyspnea, stridor, stertor and/or cyanosis, with a risk for the patients.

Only about 20% of FB included in this study were found to be Grade 0, with most dogs showing at least some degree of airway restriction. The remaining FB were considered functionally BOAS affected. In 48% of included dogs the echocardiographic examination was performed only in standing position due to the exacerbation of respiratory deficits if laterally contained.

The results of the statistical analysis showed that both methods, Cambridge classification and temperament score, and their sum were more specific than sensitive in the identification of the better containment for the echocardiographic examination. This means that they can better identify the probability that a healthy dog can be subjected to an echocardiographic examination in lateral recumbency. The obtained cutoffs for these three parameters (2.5–2.5–4.5 respectively) have never been proposed before by the literature. This study allowed us to highlight how the Cambridge classification alone is inadequate in the correct evaluation of the management of these brachycephalic dogs during the echocardiography, therefore it does not allow to discern between dogs that can be evaluated only in standing or in lateral recumbency. On the contrary, if the temperament score and the Cambridge classification are considered together in the evaluation of the dogs, it’s possible to obtain a good sensitivity (75%) and specificity (85%).

Thanks to these findings, it is possible to assume that the Cambridge classification is unlikely to fully assess the brachycephalic patient’s respiratory condition, because it does not consider important behavioral factors such as susceptibility to stress, which decisively affects the severity of clinical manifestations of this disease.

A lack of objective data on respiratory function makes it difficult to evaluate the presence, the progression, and the treatment of the disease. This consideration has important repercussions on the role of Cambridge classification for the control of surgical results: it is not able to reflect the real respiratory condition of the dog before and after the corrective surgical procedure, and therefore it cannot be considered as an objective method to compare the different surgical techniques in order to identify which of them can offer a better result in the possibility of reducing the impact of BOAS on animal welfare.

The importance of a univocal classification system lies above all in the possibility of comparing with objective methods the different corrective surgical techniques. The assessment of which technique offers the better result in the re-establishment, at least partial, of anatomical conditions associated with a satisfactory quality of breathing and, therefore, with a satisfactory quality of life, is an open challenge. To minimize the welfare impact on the increased population of affected brachycephalic dogs, and to improve management decisions, further characterization of respiratory parameters and a specific test for BOAS are required.

The present study was not without limitations. A short period of inclusion was chosen, and dogs with pathological conditions other than BOAS were excluded. Only 29 dogs were included, and studies of diagnostic performance require substantially higher number of individuals to accurately determine diagnostic accuracy. These strict inclusion/exclusion criteria contributed to reduce the size of our population. Furthermore, since the inclusion was based on the clinical findings, it did not allow the creation of sex homogeneous study groups; this constitutes a common limit to clinical research. The results showed that the gender variable could not be considered for Cambridge classification and temperament score, as well as age, weight and BCS scores. However, a larger female population could have produced different results. Furthermore, no other BOAS classification method, other than the Cambridge classification, has been used for the disease severity grading. Another limitation is the absence of a control group of dogs with no evidence of respiratory disease held in lateral recumbency to evaluate the changing of breathing.

In conclusion, results of the present study show that the Cambridge classification has low sensitivity and high specificity for predicting the type of containment during the echocardiographic examination in FB. The predictivity improves if the clinician applies the temperament score, but the best results are be obtained by combining both parameters.

Cardiologists should gain experience in standing echocardiographic examination to be able to obtain optimal evaluations in stressed or dyspneic French Bulldogs affected by BOAS. Standing position is useful and may be preferable in particular clinical situations (e.g., stressed or dyspneic patients) and the results of the present study allow the clinician to correctly evaluate the dog before containment for collateral examination. This permits to reduce the stress if the score is high enough to discourage containment. Further studies are needed to evaluate the result of the present study in a larger cohort of dogs and in different brachycephalic breeds. Comparing Cambridge classification and temperament score among canine breeds with different brachycephalic somatotype may be useful. However, we believe that our results may be useful for a proper echocardiographic containment and for screening purposes in this widespread and fashionable breed. The obtained results can be a useful tool for the clinical cardiologist who must manage these patients daily.

## Conclusions

This is the first study that evaluated if the functional grading system of the Cambridge classification and the temperament score can be useful tools in predicting the feasibility of a collateral diagnostic procedures such as echocardiography in FB affected by BOAS and of supporting stressful conditions such as lateral recumbency (generally poorly tolerated by patients with a consequent modification of the respiratory conditions and exacerbation of respiratory deficits with a risk for the patients). The results of this study demonstrated that the dog’s temperament and its susceptibility to stress added to the severity of BOAS, rather than the lonely Cambridge classification, are a good predictor of the possibility of performing the echocardiographic examination in standing position instead of lateral recumbency. Furthermore, this study underlines that BOAS is an extremely complex and difficult to evaluate condition. It can be influenced by anatomical and functional factors that can be verified and quantified, but it can be also clinically exacerbated by behavioral individual conditions, making it extremely difficult to develop a single classification system that can consider all the innumerable variables contributing to the manifestation of this syndrome. The need to develop a unique method of classification that can be used as a standard guideline for the overall management of these patients remains an open challenge.

## Supporting information

S1 Data set (XLSX)
