# Supplementary material for: Does the Cambridge classification alone predict the possibility to perform echocardiographic examination in lateral recumbency in dogs affected by brachycephalic obstructive airways syndrome?
Source: PLoS One. 2023 Jun 7;18(6):e0286914. doi: 10.1371/journal.pone.0286914 (PMC10246799; doi:10.1371/journal.pone.0286914)
Supplement: Attachment — Submitted filename: Response to Reviewers.docx [file pone.0286914.s002.docx]

PONE-D-23-09206

Does the Cambridge Classification alone predict the possibility to perform echocardiographic examination in lateral recumbency in dogs affected by Brachycephalic Obstructive Airways Syndrome?

The authors thank the Editor and the Reviewer for their thoroughly review of our study. We are very pleased about their appreciation of the work and their positive comments. Thank you very much.

We have carefully considered all reviewers’ comments and have tried to address them whenever we felt this was appropriate. We feel that the quality of our manuscript has been further improved following the Reviewers’ comments and suggestions.

Best regards

------------------------------------------------------------------------------------------------------------------------

**Review Comments to the Author**

**Reviewer #1:**

In the methodology, it is not clear whether the evaluation and classification of the scores, either by the Cambridge classification or degrees of severity of BOAS, is always carried out by the same person or if they are different people. A bias that was not mentioned in the discussion is the fact that the study was not double blind, it would be more relevant if two evaluators blinded to the results of the others classified according to the Cambridge classification or degrees of severity of BOAS to minimize the effect of subjectivity.

Thank you very much for this comment. We did not explain properly this point because we did not consider it relevant for the study focus but thank to your comment we have decided to add this information in the text. Firstly, we would like to specify that only one operator assigned the score to each dog (Doctor Federica Creta) during the clinical evaluation. Each score was than blinded checked by an experienced observer (Prof. Romussi Stefano) by the evaluation of videos. None of the score was changed by Professor, conforming the first scoring assigned by Dr. Creta. We have added this info in the materials and methods section, and we have also discussed it.

On line 133 it was said that "In the lateral recumbency, were evaluated: need of manual containment; worsening of respiratory clinical signs with the onset of polypnea, dyspnea, stridor, stertor or cyanosis; postural changes in the neck and trunk region; contraction response of the sternocleidomastoid muscles; general contraction response of the musculature with the possible appearance of tremors..." However the results of these evaluations were not demonstrated in the work, how many animals this was observed and taken into account for evaluation.

We have already discussed these data in the results section as descriptive data (lines 193-195). We have not performed a statistical analysis due to the small number of cases.

Thank you very much for your comments and suggestion, we feel that they have improved the quality of the manuscript.

------------------------------------------------------------------------------------------------------------------------

**Reviewer #2:**

The work presents an excellent contribution to the scientific community, mainly related to animal welfare during the performance of the echocardiographic examination. The research was carried out in an ethical manner, with the consent of those responsible for the bulldogs, and approval by the ethics committee for the use of animals.

Thank you very much for your appreciation of our work, we are very pleased about your decision.

------------------------------------------------------------------------------------------------------------------------
