# Supplementary material for: Does the Cambridge classification alone predict the possibility to perform echocardiographic examination in lateral recumbency in dogs affected by brachycephalic obstructive airways syndrome?
Source: PLoS One. 2023 Jun 7;18(6):e0286914. doi: 10.1371/journal.pone.0286914 (PMC10246799; doi:10.1371/journal.pone.0286914)
Supplement: Peer review history

# Peer review: Decision Letter 0

**DOI:** 10.1371/journal.pone.0286914.r001

**Authors:** Jeyasakthy Saniasiaya

2 May 2023

PONE-D-23-09206Does the Cambridge Classification alone predict the possibility to perform echocardiographic examination in lateral recumbency in dogs affected by Brachycephalic Obstructive Airways Syndrome?PLOS ONE

Dear Dr. Ghilardi,

Thank you for submitting your manuscript to PLOS ONE. After careful consideration, we feel that it has merit but does not fully meet PLOS ONE’s publication criteria as it currently stands. Therefore, we invite you to submit a revised version of the manuscript that addresses the points raised during the review process.

==============================

ACADEMIC EDITOR:

Please describe if the evaluators were blinded.

==============================

Please submit your revised manuscript by Jun 16 2023 11:59PM. If you will need more time than this to complete your revisions, please reply to this message or contact the journal office at plosone@plos.org. When you're ready to submit your revision, log on to https://www.editorialmanager.com/pone/ and select the 'Submissions Needing Revision' folder to locate your manuscript file.

Please include the following items when submitting your revised manuscript:

- A rebuttal letter that responds to each point raised by the academic editor and reviewer(s). You should upload this letter as a separate file labeled 'Response to Reviewers'.
- A marked-up copy of your manuscript that highlights changes made to the original version. You should upload this as a separate file labeled 'Revised Manuscript with Track Changes'.
- An unmarked version of your revised paper without tracked changes. You should upload this as a separate file labeled 'Manuscript'.

If you would like to make changes to your financial disclosure, please include your updated statement in your cover letter. Guidelines for resubmitting your figure files are available below the reviewer comments at the end of this letter.

If applicable, we recommend that you deposit your laboratory protocols in protocols.io to enhance the reproducibility of your results. Protocols.io assigns your protocol its own identifier (DOI) so that it can be cited independently in the future. For instructions see: https://journals.plos.org/plosone/s/submission-guidelines#loc-laboratory-protocols. Additionally, PLOS ONE offers an option for publishing peer-reviewed Lab Protocol articles, which describe protocols hosted on protocols.io. Read more information on sharing protocols at https://plos.org/protocols?utm_medium=editorial-email&utm_source=authorletters&utm_campaign=protocols.

We look forward to receiving your revised manuscript.

Kind regards,

Jeyasakthy Saniasiaya, MD, MMed ORLHNS, FEBORLHNS

Academic Editor

PLOS ONE

Journal Requirements:

When submitting your revision, we need you to address these additional requirements.

1. Please ensure that your manuscript meets PLOS ONE's style requirements, including those for file naming. The PLOS ONE style templates can be found at 

https://journals.plos.org/plosone/s/file?id=wjVg/PLOSOne_formatting_sample_main_body.pdf and 

https://journals.plos.org/plosone/s/file?id=ba62/PLOSOne_formatting_sample_title_authors_affiliations.pdf

2. Thank you for stating the following financial disclosure: 

"The funders had no role in study design, data collection and analysis, decision to publish, or preparation of the manuscript."

At this time, please address the following queries:

a) Please clarify the sources of funding (financial or material support) for your study. List the grants or organizations that supported your study, including funding received from your institution. 

b) State what role the funders took in the study. If the funders had no role in your study, please state: “The funders had no role in study design, data collection and analysis, decision to publish, or preparation of the manuscript.”

c) If any authors received a salary from any of your funders, please state which authors and which funders.

d) If you did not receive any funding for this study, please state: “The authors received no specific funding for this work.”

Please include your amended statements within your cover letter; we will change the online submission form on your behalf.

3. In your Data Availability statement, you have not specified where the minimal data set underlying the results described in your manuscript can be found. PLOS defines a study's minimal data set as the underlying data used to reach the conclusions drawn in the manuscript and any additional data required to replicate the reported study findings in their entirety. All PLOS journals require that the minimal data set be made fully available. For more information about our data policy, please see http://journals.plos.org/plosone/s/data-availability.

Upon re-submitting your revised manuscript, please upload your study’s minimal underlying data set as either Supporting Information files or to a stable, public repository and include the relevant URLs, DOIs, or accession numbers within your revised cover letter. For a list of acceptable repositories, please see http://journals.plos.org/plosone/s/data-availability#loc-recommended-repositories. Any potentially identifying patient information must be fully anonymized.

Important: If there are ethical or legal restrictions to sharing your data publicly, please explain these restrictions in detail. Please see our guidelines for more information on what we consider unacceptable restrictions to publicly sharing data: http://journals.plos.org/plosone/s/data-availability#loc-unacceptable-data-access-restrictions. Note that it is not acceptable for the authors to be the sole named individuals responsible for ensuring data access.

We will update your Data Availability statement to reflect the information you provide in your cover letter.

4. We note that you have referenced (ie. Bewick et al. [5]) which has currently not yet been accepted for publication. Please remove this from your References and amend this to state in the body of your manuscript: (ie “Bewick et al. [Unpublished]”) as detailed online in our guide for authors

http://journals.plos.org/plosone/s/submission-guidelines#loc-reference-style

5. Please review your reference list to ensure that it is complete and correct. If you have cited papers that have been retracted, please include the rationale for doing so in the manuscript text, or remove these references and replace them with relevant current references. Any changes to the reference list should be mentioned in the rebuttal letter that accompanies your revised manuscript. If you need to cite a retracted article, indicate the article’s retracted status in the References list and also include a citation and full reference for the retraction notice.

Additional Editor Comments:

Please describe if the evaluators were blinded

[Note: HTML markup is below. Please do not edit.]

Reviewers' comments:

Reviewer's Responses to Questions

**Comments to the Author**

1. Is the manuscript technically sound, and do the data support the conclusions?

The manuscript must describe a technically sound piece of scientific research with data that supports the conclusions. Experiments must have been conducted rigorously, with appropriate controls, replication, and sample sizes. The conclusions must be drawn appropriately based on the data presented. 

Reviewer #1: Yes

Reviewer #2: Yes

**********

2. Has the statistical analysis been performed appropriately and rigorously? 

Reviewer #1: I Don't Know

Reviewer #2: Yes

**********

3. Have the authors made all data underlying the findings in their manuscript fully available?

The PLOS Data policy requires authors to make all data underlying the findings described in their manuscript fully available without restriction, with rare exception (please refer to the Data Availability Statement in the manuscript PDF file). The data should be provided as part of the manuscript or its supporting information, or deposited to a public repository. For example, in addition to summary statistics, the data points behind means, medians and variance measures should be available. If there are restrictions on publicly sharing data—e.g. participant privacy or use of data from a third party—those must be specified.

Reviewer #1: No

Reviewer #2: Yes

**********

4. Is the manuscript presented in an intelligible fashion and written in standard English?

PLOS ONE does not copyedit accepted manuscripts, so the language in submitted articles must be clear, correct, and unambiguous. Any typographical or grammatical errors should be corrected at revision, so please note any specific errors here.

Reviewer #1: Yes

Reviewer #2: Yes

**********

5. Review Comments to the Author

Please use the space provided to explain your answers to the questions above. You may also include additional comments for the author, including concerns about dual publication, research ethics, or publication ethics. (Please upload your review as an attachment if it exceeds 20,000 characters)

Reviewer #1: In the methodology, it is not clear whether the evaluation and classification of the scores, either by the Cambridge classification or degrees of severity of BOAS, is always carried out by the same person or if they are different people. A bias that was not mentioned in the discussion is the fact that the study was not double blind, it would be more relevant if two evaluators blinded to the results of the others classified according to the Cambridge classification or degrees of severity of BOAS to minimize the effect of subjectivity.

On line 133 it was said that "In the lateral recumbency, were evaluated: need of manual containment; worsening of respiratory clinical signs with the onset of polypnea, dyspnea, stridor, stertor or cyanosis; postural changes in the neck and trunk region; contraction response of the sternocleidomastoid muscles; general contraction response of the musculature with the possible appearance of tremors..." However the results of these evaluations were not demonstrated in the work, how many animals this was observed and taken into account for evaluation.

Reviewer #2: The work presents an excellent contribution to the scientific community, mainly related to animal welfare during the performance of the echocardiographic examination. The research was carried out in an ethical manner, with the consent of those responsible for the bulldogs, and approval by the ethics committee for the use of animals.

**********

6. PLOS authors have the option to publish the peer review history of their article (what does this mean?). If published, this will include your full peer review and any attached files.

If you choose “no”, your identity will remain anonymous but your review may still be made public.

**Do you want your identity to be public for this peer review?** For information about this choice, including consent withdrawal, please see our Privacy Policy.

Reviewer #1: **Yes: **Bruno Alberigi

Reviewer #2: No

**********

[NOTE: If reviewer comments were submitted as an attachment file, they will be attached to this email and accessible via the submission site. Please log into your account, locate the manuscript record, and check for the action link "View Attachments". If this link does not appear, there are no attachment files.]

While revising your submission, please upload your figure files to the Preflight Analysis and Conversion Engine (PACE) digital diagnostic tool, https://pacev2.apexcovantage.com/. PACE helps ensure that figures meet PLOS requirements. To use PACE, you must first register as a user. Registration is free. Then, login and navigate to the UPLOAD tab, where you will find detailed instructions on how to use the tool. If you encounter any issues or have any questions when using PACE, please email PLOS at figures@plos.org. Please note that Supporting Information files do not need this step.

# Author comment: Author response to Decision Letter 0

**DOI:** 10.1371/journal.pone.0286914.r002

16 May 2023

PONE-D-23-09206

Does the Cambridge Classification alone predict the possibility to perform echocardiographic examination in lateral recumbency in dogs affected by Brachycephalic Obstructive Airways Syndrome?

The authors thank the Editor and the Reviewer for their thoroughly review of our study. We are very pleased about their appreciation of the work and their positive comments. Thank you very much.

We have carefully considered all reviewers’ comments and have tried to address them whenever we felt this was appropriate. We feel that the quality of our manuscript has been further improved following the Reviewers’ comments and suggestions.

Best regards

------------------------------------------------------------------------------------------------------------------------

Review Comments to the Author

Reviewer #1: 

In the methodology, it is not clear whether the evaluation and classification of the scores, either by the Cambridge classification or degrees of severity of BOAS, is always carried out by the same person or if they are different people. A bias that was not mentioned in the discussion is the fact that the study was not double blind, it would be more relevant if two evaluators blinded to the results of the others classified according to the Cambridge classification or degrees of severity of BOAS to minimize the effect of subjectivity.

Thank you very much for this comment. We did not explain properly this point because we did not consider it relevant for the study focus but thank to your comment we have decided to add this information in the text. Firstly, we would like to specify that only one operator assigned the score to each dog (Doctor Federica Creta) during the clinical evaluation. Each score was than blinded checked by an experienced observer (Prof. Romussi Stefano) by the evaluation of videos. None of the score was changed by Professor, conforming the first scoring assigned by Dr. Creta. We have added this info in the materials and methods section, and we have also discussed it. 

On line 133 it was said that "In the lateral recumbency, were evaluated: need of manual containment; worsening of respiratory clinical signs with the onset of polypnea, dyspnea, stridor, stertor or cyanosis; postural changes in the neck and trunk region; contraction response of the sternocleidomastoid muscles; general contraction response of the musculature with the possible appearance of tremors..." However the results of these evaluations were not demonstrated in the work, how many animals this was observed and taken into account for evaluation.

We have already discussed these data in the results section as descriptive data (lines 193-195). We have not performed a statistical analysis due to the small number of cases.

Thank you very much for your comments and suggestion, we feel that they have improved the quality of the manuscript. 

------------------------------------------------------------------------------------------------------------------------

Reviewer #2: 

The work presents an excellent contribution to the scientific community, mainly related to animal welfare during the performance of the echocardiographic examination. The research was carried out in an ethical manner, with the consent of those responsible for the bulldogs, and approval by the ethics committee for the use of animals.

Thank you very much for your appreciation of our work, we are very pleased about your decision. 

------------------------------------------------------------------------------------------------------------------------

Attachment Submitted filename: Response to Reviewers.docx Click here for additional data file.

# Peer review: Decision Letter 1

**DOI:** 10.1371/journal.pone.0286914.r003

**Authors:** Jeyasakthy Saniasiaya

26 May 2023

Does the Cambridge Classification alone predict the possibility to perform echocardiographic examination in lateral recumbency in dogs affected by Brachycephalic Obstructive Airways Syndrome?

PONE-D-23-09206R1

Dear Dr. Ghilardi,

We’re pleased to inform you that your manuscript has been judged scientifically suitable for publication and will be formally accepted for publication once it meets all outstanding technical requirements.

Within one week, you’ll receive an e-mail detailing the required amendments. When these have been addressed, you’ll receive a formal acceptance letter and your manuscript will be scheduled for publication.

An invoice for payment will follow shortly after the formal acceptance. To ensure an efficient process, please log into Editorial Manager at http://www.editorialmanager.com/pone/, click the 'Update My Information' link at the top of the page, and double check that your user information is up-to-date. If you have any billing related questions, please contact our Author Billing department directly at authorbilling@plos.org.

If your institution or institutions have a press office, please notify them about your upcoming paper to help maximize its impact. If they’ll be preparing press materials, please inform our press team as soon as possible -- no later than 48 hours after receiving the formal acceptance. Your manuscript will remain under strict press embargo until 2 pm Eastern Time on the date of publication. For more information, please contact onepress@plos.org.

Kind regards,

Jeyasakthy Saniasiaya, MD, MMed ORLHNS, FEBORLHNS

Academic Editor

PLOS ONE

Additional Editor Comments (optional):

Authors have addressed all suggestions adequately

Reviewers' comments:

Reviewer's Responses to Questions

**Comments to the Author**

Reviewer #1: All comments have been addressed

Reviewer #2: All comments have been addressed

**********

2. Is the manuscript technically sound, and do the data support the conclusions?

The manuscript must describe a technically sound piece of scientific research with data that supports the conclusions. Experiments must have been conducted rigorously, with appropriate controls, replication, and sample sizes. The conclusions must be drawn appropriately based on the data presented. 

Reviewer #1: Yes

Reviewer #2: Yes

**********

3. Has the statistical analysis been performed appropriately and rigorously? 

Reviewer #1: Yes

Reviewer #2: Yes

**********

4. Have the authors made all data underlying the findings in their manuscript fully available?

The PLOS Data policy requires authors to make all data underlying the findings described in their manuscript fully available without restriction, with rare exception (please refer to the Data Availability Statement in the manuscript PDF file). The data should be provided as part of the manuscript or its supporting information, or deposited to a public repository. For example, in addition to summary statistics, the data points behind means, medians and variance measures should be available. If there are restrictions on publicly sharing data—e.g. participant privacy or use of data from a third party—those must be specified.

Reviewer #1: Yes

Reviewer #2: Yes

**********

5. Is the manuscript presented in an intelligible fashion and written in standard English?

PLOS ONE does not copyedit accepted manuscripts, so the language in submitted articles must be clear, correct, and unambiguous. Any typographical or grammatical errors should be corrected at revision, so please note any specific errors here.

Reviewer #1: Yes

Reviewer #2: Yes

**********

6. Review Comments to the Author

Please use the space provided to explain your answers to the questions above. You may also include additional comments for the author, including concerns about dual publication, research ethics, or publication ethics. (Please upload your review as an attachment if it exceeds 20,000 characters)

Reviewer #1: (No Response)

Reviewer #2: all recommendations were met. For this reason I recommend that the article be accepted for publication.

**********

7. PLOS authors have the option to publish the peer review history of their article (what does this mean?). If published, this will include your full peer review and any attached files.

If you choose “no”, your identity will remain anonymous but your review may still be made public.

**Do you want your identity to be public for this peer review?** For information about this choice, including consent withdrawal, please see our Privacy Policy.

Reviewer #1: **Yes: **Bruno Alberigi

Reviewer #2: **Yes: **Alexandre Bendas

**********

# Editor report: Acceptance letter

**DOI:** 10.1371/journal.pone.0286914.r004

**Authors:** Jeyasakthy Saniasiaya

30 May 2023

PONE-D-23-09206R1 

Does the Cambridge Classification alone predict the possibility to perform echocardiographic examination in lateral recumbency in dogs affected by Brachycephalic Obstructive Airways Syndrome? 

Dear Dr. Ghilardi:

I'm pleased to inform you that your manuscript has been deemed suitable for publication in PLOS ONE. Congratulations! Your manuscript is now with our production department. 

If your institution or institutions have a press office, please let them know about your upcoming paper now to help maximize its impact. If they'll be preparing press materials, please inform our press team within the next 48 hours. Your manuscript will remain under strict press embargo until 2 pm Eastern Time on the date of publication. For more information please contact onepress@plos.org.

If we can help with anything else, please email us at plosone@plos.org. 

Thank you for submitting your work to PLOS ONE and supporting open access. 

Kind regards, 

PLOS ONE Editorial Office Staff

on behalf of

Dr. Jeyasakthy Saniasiaya 

Academic Editor

PLOS ONE
